# A potential neuromodulation target for PTSD in Veterans derived from focal brain lesions

**DOI:** 10.21203/rs.3.rs-3132332/v1

**Published:** 2024-03-19

**Authors:** Shan Siddiqi, Noah S. Philip, Stephan Palm, Amanda Arulpragasam, Jennifer Barredo, Heather Bouchard, Michael Ferguson, Jordan Grafman, Rajendra Morey, Michael Fox, David Carreon

**Affiliations:** Harvard Medical School, Brigham & Women’s Hospital; Alpert Medical School of Brown University, Center for Neurorestoration and Neurotechnology, Providence VA Medical Center; Brigham & Women’s Hospital; Brown University Alpert School of Medicine; Brown University Alpert School of Medicine; Duke University School of Medicine; Harvard Medical School; Shirley Ryan AbilityLab; Duke University School of Medicine; Brigham and Women’s Hospital, Harvard Medical School; Acacia Mental Health

## Abstract

Neuromodulation trials for PTSD have yielded mixed results, and the optimal neuroanatomical target remains unclear. We analyzed three datasets to study brain circuitry causally linked to PTSD in military Veterans. After penetrating traumatic brain injury (n=193), lesions that reduced probability of PTSD were preferentially connected to a circuit including the medial prefrontal cortex (mPFC), amygdala, and anterolateral temporal lobe (cross-validation p=0.01). In Veterans without lesions (n=180), PTSD was specifically associated with connectivity within this circuit (p<0.01). Connectivity change within this circuit correlated with PTSD improvement after transcranial magnetic stimulation (TMS) (n=20) (p<0.01), even though the circuit was not directly targeted. Finally, we directly targeted this circuit with fMRI-guided accelerated TMS, leading to rapid resolution of symptoms in a patient with severe lifelong PTSD. All results were independent of depression severity. This lesion-based PTSD circuit may serve as a neuromodulation target for Veterans with PTSD.

## Introduction

Posttraumatic stress disorder (PTSD) afflicts up to 30% of trauma survivors and military combat Veterans^1,2^, and is associated with disabling comorbidities such as depression, substance use, and suicidality^3^. Medications and psychotherapy are only modestly effective^4,5^, so targeted neuromodulation has been proposed as a new treatment approach, building on its established efficacy for depression^6–8^. Neuromodulation techniques are designed to activate or inhibit brain circuits that are causally implicated in any given symptom or disorder^7–9^. For instance, focal modulation of the amygdala with deep brain stimulation (DBS)^8^, laser ablation^10^, and responsive neurostimulation^11^ has shown preliminary efficacy for PTSD in case reports, consistent with the finding that incidental lesions to the amygdala can reduce probability of PTSD^12^. Similar results have been seen with DBS to the uncinate fasciculus, which connects the amygdala to the ventromedial prefrontal cortex^13,14^. However, these invasive procedures may not be appropriate for many patients.

Noninvasive modulation of brain circuitry is possible using transcranial magnetic stimulation (TMS), but TMS cannot directly access deep targets such as the amygdala. Instead, many TMS trials for PTSD have targeted the dorsolateral prefrontal cortex (DLPFC), an effective target for major depression^7,15^. However, PTSD may not respond to the same targets as depression^16^. Two multi-center clinical trials suggest that effective antidepressant targets may even worsen PTSD symptoms (relative to sham)^17,18^. These TMS results were unexpected, but are consistent with animal models showing that stimulation to specific circuits under specific conditions can exacerbate fear^19^. Thus, it is important to identify treatment targets specific for PTSD.

Importantly, the TMS target for depression in humans was inspired partly by studies showing that lesions to the left DLPFC can increase depression risk^20^. More recently, we showed that effective TMS and DBS targets for depression are connected to the same brain circuit as lesions that increase depression risk^21^.

A causal lesion-derived circuit model of PTSD may similarly reveal improved treatment targets^9,22^. Thus, we sought to identify TMS targets based on connectivity of lesions that affect PTSD symptoms, independently of depression. First, in 193 military Veterans with penetrating traumatic brain injury (TBI), we derived and cross-validated a brain circuit connected to lesions that reduce probability of developing PTSD. Next, in 180 Veterans without brain lesions, we assessed whether connectivity in this circuit was associated with PTSD. Finally, in 20 Veterans who received active or sham TMS, we assessed whether TMS-induced change in this circuit correlated with improvement in PTSD severity.

## Results

### Reduced PTSD prevalence after penetrating head trauma

First, in the Vietnam Head Injury Study^23^, we compared Veterans with penetrating head trauma (n=196) versus Veterans without brain injury (n=55) with similar age and combat exposure based on military records. The primary outcome was PTSD lifetime prevalence on the Structured Clinical Interview for DSM-IV-TR Axis I disorders, Non-Patient Edition (SCID), which is the gold standard for psychiatric diagnosis^24^ and reliably isolates PTSD versus other comorbidities^12,23^. Participants were given a PTSD score of 0, 1, or 2, corresponding to no PTSD, subthreshold PTSD, or meeting full criteria for PTSD. A positive score was defined as having “PTSD symptoms,” and meeting full criteria was defined as “PTSD diagnosis.” Three brain-injured participants and one control participant were excluded due to incomplete SCID.

Building on prior reports showing that certain lesions can protect against PTSD^12^, we found that penetrating head trauma in general was associated with lower lifetime prevalence of PTSD symptoms (52% versus 70%, p<0.05) and PTSD diagnosis (32% versus 47%, p<0.05). Other similarities and differences between groups are summarized in Table S1.

### Relationship between lesion location and PTSD

**Figure 1:**
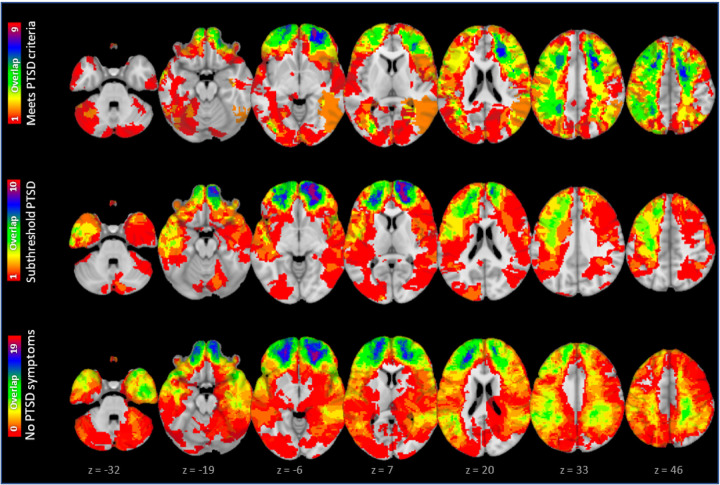
61 participants met criteria for PTSD, 39 developed subthreshold PTSD symptoms, and 93 had neither. Lesion overlap from all three groups is depicted here.

61 participants developed PTSD, 39 developed subthreshold PTSD symptoms, and 93 had neither, with heterogeneity in lesion location (Fig 1). Consistent with prior work^12^, PTSD score was lower for patients with amygdala lesions (n=15) than other lesions (n=178) (p=0.009). However, 4 patients with amygdala lesions still had subthreshold PTSD symptoms and most patients without PTSD symptoms had lesions outside the amygdala (n=82).

### A brain circuit that protects against PTSD

**Figure 2:**
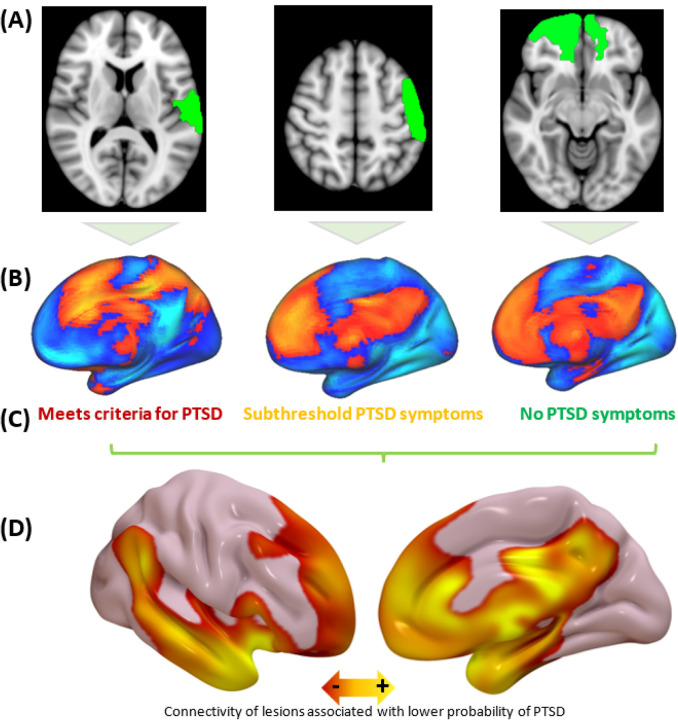
**(a)** Each participant’s lesion was localized and mapped to a common MNI template (three examples shown here). **(b)** Using a normative connectome database (n=1000), we mapped the estimated connectivity profile of each lesion. **(c)** Across all participants, PTSD status was compared with lesion connectivity in order to identify which connections are associated with PTSD. **(d)** PTSD prevalence was lower in participants whose lesions were more connected to a network of regions that included the amygdala and the medial prefrontal cortex.

Next, we used lesion network mapping (LNM) to derive a circuit connected to lesions that modify the probability of developing PTSD symptoms. This method uses a normative connectome database (n=1000) generated from resting-state functional connectivity (rsFC) data to estimate whole-brain connectivity of each lesion location^25^. Each lesion was mapped to a standard template brain (Fig. 2a). Each lesion’s connectivity profile (Fig. 2b) was compared with PTSD status to map connections associated with PTSD (Fig. 2c) using partial Pearson correlation at each voxel. Depression, measured using sum score on the Beck Depression Inventory (BDI) second edition, was included as a covariate. Lesions associated with lower prevalence of PTSD were connected to the mPFC, anterolateral temporal lobe, and medial temporal lobe, including the hippocampus and amygdala (Fig. 2d). We refer to this connectivity map as our “PTSD circuit.” The peak in this PTSD circuit was in the tapetum of the corpus callosum (Fig. S2a) (p_FWE_<0.05), which connects the two medial temporal lobes to each other^26^. Because our goal was to identify TMS targets near the cortical surface, we focused on the full circuit topography rather than just one peak region, as in our prior work^9^.

**Figure 3:**
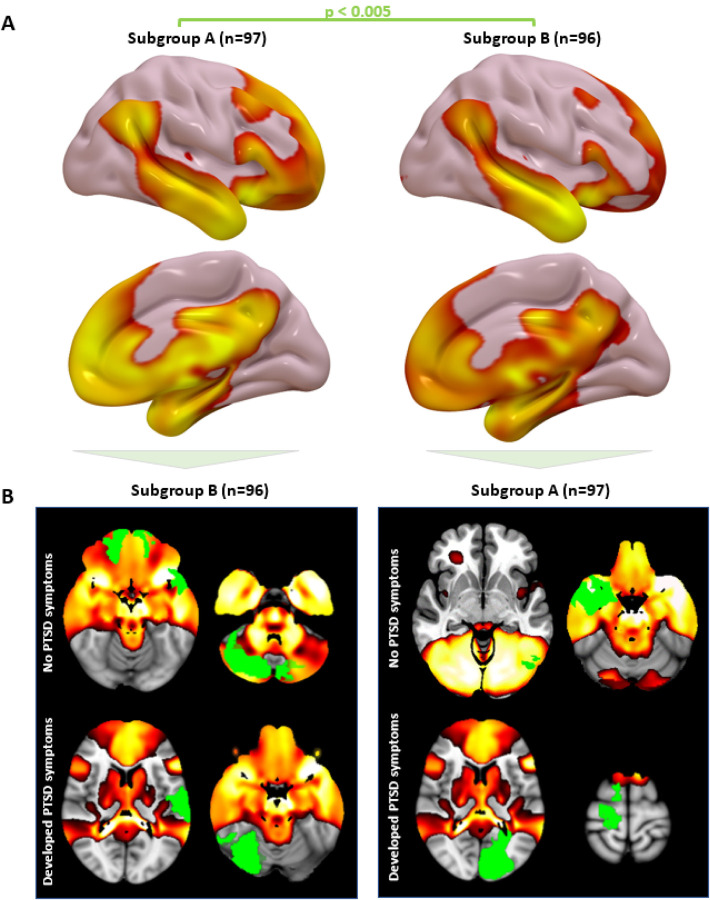
Split-half cross-validation confirmed that the PTSD circuit predicts clinical variance in an independent sample. **(a)** When the dataset was split into two subgroups, each subgroup yielded similar maps, even with 1000 randomly-sampled group assignments (mean spatial r=0.57). **(b)** Lesions in one subgroup were compared with a PTSD circuit derived from the other subgroup. The degree of overlap was significantly associated with PTSD status (p=0.01).

To test for significance of this topography, we split the dataset into two subgroups and re-generated the circuit in each subgroup. Across 10,000 different randomly-sampled subgroup assignments, both subgroups yielded similar PTSD circuits (Fig. 3a, mean spatial r=0.57, p<0.05). This spatial correlation means that 32% of the variance in the spatial topography of one circuit can be predicted from the topography of the other circuit, while the accompanying p-value means that this degree of similarity is higher than expected by chance when each patient’s clinical outcome is randomly shuffled with a different patient’s lesion location (10,000 permutations). PTSD scores in each subgroup could be predicted by lesion overlap with a PTSD circuit generated from the other subgroup (p<0.01, 10,000 randomly-sampled iterations) (Fig. 3b). Of note, negative values in the map were also identified (Fig. S2b) but did not pass cross-validation (p=0.8). The split-half spatial correlation (r=0.57) was stronger than the correlation of the PTSD circuit with the default mode network (DMN) (r=0.36), the limbic network (r=0.25), or the other canonical networks as defined by Yeo et al (r<0.13)^27^, demonstrating that the PTSD circuit looks more like itself than like other networks.

We next assessed whether our results were driven by any individual brain region or a circuit-level phenomenon. We repeated the primary analysis after excluding all 15 amygdala lesions, yielding a nearly-identical map (spatial r=0.91) which again predicted PTSD score on split-half cross-validation (p<0.05). We also repeated this analysis for all 246 brain regions in the Brainnetome atlas^28^, yielding a highly similar map in all cases (median r=0.99, range 0.88–0.999). Next, we used voxel lesion symptom mapping (VLSM)^28,29^ to identify other PTSD-associated locations across the whole brain. Multiple lesion locations were marginally associated with PTSD (Fig. S1), but these associations were not stronger than chance (p_FWE_=0.24). The overall VLSM map did not survive cross-validation using spatial correlation (split-half spatial r=0.02) or prediction of PTSD score (split-half r=0.07, p=0.33; leave-one-out r=0.03, p=0.63). Thus, in a whole-brain data-driven analysis, we did not detect any individual locations associated with PTSD. Of note, no lesions directly intersected the peak location in the tapetum, further illustrating a network-level phenomenon.

We conducted additional analyses to confirm that this result was not biased by our choice of outcome metrics. A nearly-identical circuit was generated when excluding 50 patients with subthreshold PTSD (spatial r=0.999, Fig. S3a) or when using continuous PTSD severity as measured by the Clinician Assessment for PTSD Symptoms (CAPS) (r=0.96) (Fig. S3b). The circuit was not driven by comorbidities such as alcoholism, cognitive impairment, or anxiety, as nearly-identical PTSD circuits were generated when controlling for McAndrews alcoholism risk score (spatial r=0.98, Fig. S3b)^30^, Folstein mini-mental state exam score (spatial r=0.96, Fig. S3c), total number of anxiety disorders on the SCID (spatial r=0.98), or current anxiety on the neurobehavioral rating scale (r=0.98). A nearly-identical PTSD circuit was also generated when not controlling for depression (spatial r=0.96, Fig. S3d). The circuit was not driven by any individual symptom, as nearly-identical maps were generated from the CAPS subscales for “avoidance/numbing,” “re-experiencing,” and “distress” (r>0.94), while overall similar circuits were generated from the remaining subscales (mean r=0.88, range 0.65–0.96) (Fig. S4). Weaker associations (p=0.001) were seen with depressive symptoms on the BDI (mean r=0.39, range −0.23–0.82) (Fig. S4).

### Generalizability to patients without brain lesions

To evaluate generalizability beyond lesions, we compared our lesion-derived PTSD circuit to a map of published PTSD-related neuroimaging findings from Neurosynth, which automatically synthesizes the neuroimaging literature associated with any given search term^31^. Our lesion-derived PTSD circuit overlapped significantly with the Neurosynth PTSD map (p<0.01, 10,000 permutations), but not with the Neurosynth maps for depression (p=0.55) or anxiety (p=0.08). This difference was significant for depression (p<0.05, 10,000 permutations), but not anxiety (p=0.4). This suggests that our lesion-based PTSD circuit is consistent with the published neuroimaging correlates of PTSD.

**Figure 4:**
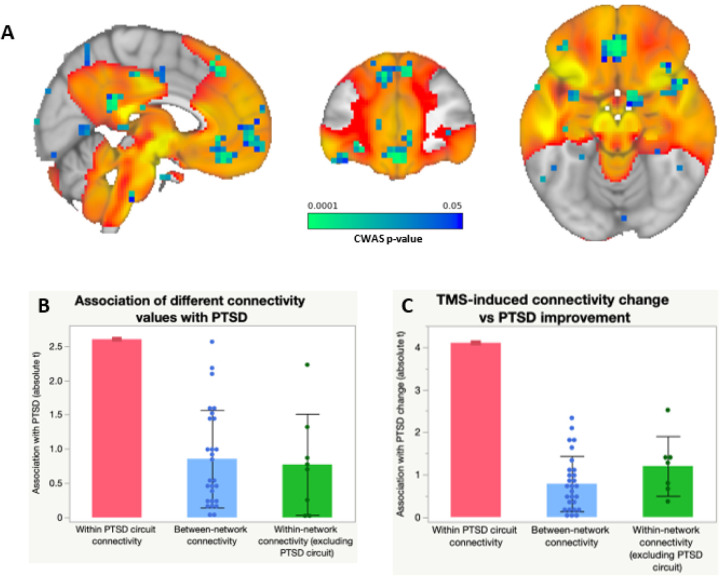
Individualized connectivity within the PTSD circuit is abnormal in PTSD patients. **(a)** CWAS showed that voxels whose connectivity was abnormal in PTSD patients (blue/green) were significantly more likely to be within the lesion-derived PTSD circuit than outside the circuit. **(b)** PTSD symptoms were associated with abnormal connectivity within our circuit. This effect was stronger for the PTSD circuit than within or between seven canonical resting-state networks. Note: absolute t-values are depicted here to illustrate the overall strength of associations in each group **(c)** TMS-induced change in PTSD severity was associated with TMS-induced change within our circuit, but not other networks. Note: absolute t-values are depicted here to illustrate the overall strength of association in each group.

We also evaluated generalizability in an independent dataset of rsFC from 180 Veterans without lesions, including n=62 with PTSD. We conducted a connectome-wide association study (CWAS) using multivariate distance matrix regression^32^ to identify voxels whose rsFC is most abnormal in PTSD patients, controlling for TBI and depression. PTSD-associated voxels (detection threshold p<0.01) were more likely to be inside our circuit than outside the circuit (Odds Ratio=14.7, p=0.01, 10,000 permutations) (Fig. 4a). This result remained significant when using different detection thresholds of p<0.05 or p<0.001 for CWAS. To test for anatomical specificity, this analysis was repeated for seven canonical networks as defined by Yeo et al^27^. Only the DMN contained a significant proportion of PTSD-associated voxels (OR=7.4, p=0.01), but this was weaker than the proportion of PTSD-associated voxels within our lesion-based PTSD circuit (p=0.04).

To further assess specificity and demonstrate concordance across methods, we used our lesion-based circuit as a weighted seed to assess within-network rsFC, as in our prior work^33,34^. Increased rsFC in this circuit was associated with reduced PTSD prevalence, whether controlling for depression (t=2.60, p=0.01) (Fig. S5a) or not (t=2.31, p=0.02). This result was unchanged when controlling for comorbid generalized anxiety disorder (t=2.59, p=0.01) or any of the 17 items on the Davidson Trauma Scale (p<0.05), including the anxiety item. PTSD was more associated with rsFC within our circuit than within or between any of the control networks (Fig. 4b)^27^.

To evaluate relevance for TMS, we assessed rsFC within our circuit in 20 Veterans who participated in a sham-controlled trial of TMS to the right DLPFC for PTSD^7^. Symptom severity was quantified using the PTSD Checklist for DSM-5 (PCL-5). Decreased rsFC within our circuit was correlated with reduction in PTSD severity with active versus sham TMS (Group × Connectivity change interaction), whether controlling for depression (t=4.1, p=0.001) (Fig. S5b) or not (t=3.4, p=0.004). This effect was anatomically specific to our PTSD circuit, which showed a stronger association than connectivity within or between any of the seven Yeo networks (p<0.05 in all cases) (Fig. 4c)^27^. The effect was also behaviorally specific to PTSD versus overall anxiety, as the result was unchanged when adding change in state-trait anxiety inventory as a covariate (t=3.3, p=0.007).

Decreased rsFC between the right DLPFC stimulation site and our circuit was also correlated with reduction in PTSD severity, whether controlling for depression (t=−3.5, p=0.003) or not (t=−2.3, p=0.03). This effect was stronger than rsFC between the stimulation region and any of the seven Yeo control networks (p<0.05 in all cases).

### Exploring Relevance for guiding TMS for PTSD

Finally, we explored whether our lesion-based circuit for PTSD might be relevant for guiding where and how to administer TMS treatment, based on alignment with the existing literature.

**Figure 5:**
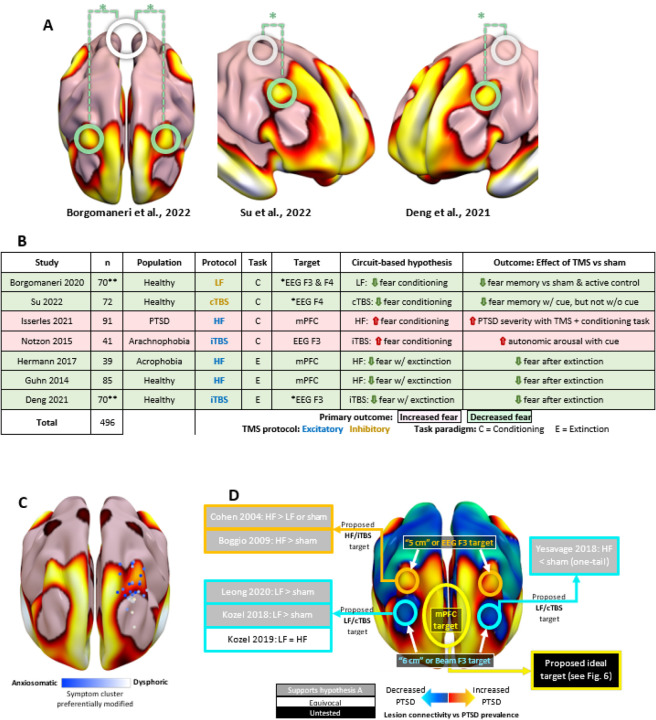
Comparison to published TMS targets. **(a)** Three head-to-head trials conducted a direct comparison between EEG F3/F4 targets (which incidentally fell within our PTSD circuit) and sites that incidentally fell outside the circuit. All three of these trials found a significant advantage to stimulating the PTSD circuit. **(b)** Two studies reported increased fear when a fear conditioning task was paired with excitatory stimulation to our circuit. Five studies reported reduced fear when a fear conditioning task was paired with inhibitory stimulation or a fear extinction task was paired with excitatory stimulation. *Head-to-head comparison of TMS to the PTSD circuit vs. other circuits. **Results pooled across multiple groups/experiments that met inclusion criteria. **(c)** The PTSD circuit was highly similar to the previously-published circuit that is connected to TMS sites associated with improvement in “anxiosomatic” symptoms (blue) versus sites associated with improvement in dysphoric symptoms (white). **(d)** Amongst commonly-used TMS targets, the “5cm” and EEG F3/4 targets were positively correlated with our circuit, while the “6cm” and Beam F3/F4 targets were negatively correlated with our circuit. The peak TMS target likely to modify PTSD symptoms was in the mPFC. We identified six prior studies with five different stimulation targets in which TMS was applied with an extinction task or no task (yellow = positively connected targets, blue = negatively connected targets). Five of these studies were consistent with Hypothesis A, no studies were consistent with Hypothesis B, and one study was equivocal. LF = Low-Frequency TMS, HF = High-frequency TMS, MDD = major depressive disorder, iTBS = intermittent theta burst stimulation, cTBS = continuous theta burst stimulation

First, we explored whether the topography of our PTSD circuit might inform where to administer TMS. We identified three trials that performed a head-to-head comparison of different TMS targets for modulating fear conditioning or fear extinction. In all three studies, TMS-induced changes in fear were greater when stimulating areas that fall within our PTSD circuit (EEG F3 and F4 sites, localized as previously reported^16^) relative to areas that did not fall within our circuit (Figure 5a).

Second, we explored whether our lesion-based PTSD circuit might inform *how* to administer TMS. Different protocols are believed to be excitatory versus inhibitory, and are often used in an effort to upregulate or downregulate brain circuits, respectively. These protocols can be combined with either a fear conditioning or fear extinction task, leading to many potential combinations. To test how these different combinations affect fear, we identified seven well-powered randomized trials that employed conditioning or extinction tasks with different TMS protocols, mostly in healthy controls. Because our circuit was derived from lesions (presumably inhibitory) that decrease the effects of a traumatic event (fear conditioning), one might expect that inhibitory TMS to our circuit would reduce fear conditioning, a hypothesis consistent with the results of the two trials that tested this combination (Fig. 5b)^35,36^. Conversely, one might expect that excitatory TMS paired with a fear conditioning task might increase PTSD severity or cue-induced autonomic arousal, which was the (unanticipated) result in two other trials^18,37^. Finally, we would expect excitatory stimulation to our circuit to potentiate the effects of a fear extinction task, consistent with the results of three more studies^38–40^. Across all seven studies, our lesion-based hypothesis was consistent with TMS-induced improvement or worsening in fear.

Third, we explored whether our PTSD circuit aligns with TMS sites that modulate “anxiosomatic” versus “dysphoric” symptoms in patients with MDD such as sexual dysfunction, irritability, sleep disturbances, past failure, and indecisiveness. In both datasets that collected the BDI (n=373), PTSD diagnosis was more correlated with anxiosomatic symptoms than dysphoric symptoms (r=0.33 vs 0.19, p<10^−5^). TMS sites that were more connected to the PTSD circuit were more likely to improve anxiosomatic symptoms (n=111, r=0.22, p=0.018) but not overall depression scores (n=111, r=−0.08, p=0.38). Our previously published “anxiosomatic circuit” derived from these TMS data showed similar topography to our PTSD circuit (spatial r=0.62, p<0.05, 10,000 permutations) (Fig. 5c).

To identify potential TMS targets, we mapped regions that were positively and negatively connected to our lesion-derived PTSD circuit (Fig. 5d), with a focus on cortical regions accessible with TMS. We then explored how this map aligns with the results of prior TMS clinical trials for PTSD. In contrast to our earlier analysis focused on fear effects when TMS is combined with tasks, most TMS trials for PTSD were conducted without a task. We identified six prior TMS trials with no concurrent fear-related task in patients with PTSD (Fig. 5e). We hypothesized that the absence of any psychological task would be similar to a spontaneous extinction condition^41–43^. As such, one would expect that positive values in our circuit would represent better excitatory TMS targets, while negative values would represent better inhibitory targets (hypothesis A). However it is possible that the opposite is true, in which case negative values would represent better excitatory targets, while positive values would represent better inhibitory targets (hypothesis B). We found that 5 of 6 studies supported hypothesis A, no studies supported hypothesis B (z-test for proportions p=0.004), and the sixth was equivocal (Fig. 5d). The peak positive connection was in the mPFC at MNI coordinates [−12,66,24], which we predict would be an optimal PTSD target if administering excitatory TMS in the absence of task.

### Case example of TMS targeted to the PTSD circuit

To illustrate how the PTSD circuit might be used clinically, we present the case of a 62-year-old man with treatment-resistant PTSD due to childhood physical abuse who was referred by his primary psychiatrist for consideration of clinical TMS. The patient rated his PTSD severity as “10/10” and baseline PCL-5 score was 70/80 (severe PTSD). He also struggled with migraines and palpitations, particularly in association with PTSD triggers. Other clinical characteristics and imaging/treatment parameters are in Box S1. An extensive informed consent process was conducted to discuss the risks and benefits of this novel off-label target, including the possibility of unexpected adverse effects or exacerbation of symptoms. The patient also provided informed consent for publication of this case report. Analysis of clinical outcomes data was approved by Pearl institutional review board (22-ACAC-101).

**Figure 6:**
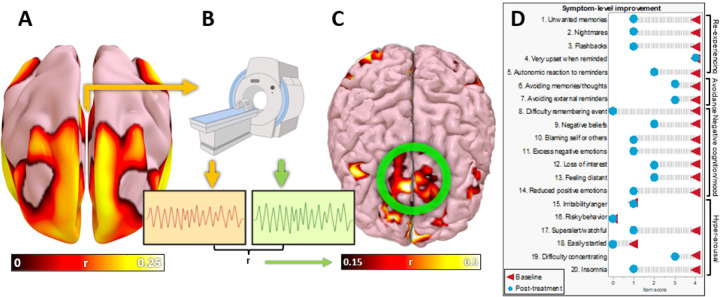
Personalizing and targeting the PTSD network with TMS. The lesion-derived PTSD circuit **(a)** was personalized using a resting-state fMRI scan **(b)**, in which the timecourse was extracted from the PTSD circuit (orange) and from other voxels in the brain (green). Timecourses with the strongest correlation to the PTSD circuit were identified, and are plotted here on a 3D reconstruction of the patient’s own brain **(c)**. The peak voxel cluster on the prefrontal surface (circled in green) was chosen as a treatment target. **(d)** After 50 sessions of TMS treatment, the participant reported dramatic improvement, including persistent improvement in 17 of 20 PTSD symptoms across all four symptom domains.

To identify a personalized TMS target, we combined our group-level PTSD circuit (Fig. 6a) with resting-state fMRI data from the patient (Fig. 6b). The mean resting-state timecourse from the PTSD circuit was compared with every other voxel using Pearson correlations, yielding an individualized PTSD circuit (Fig. 6c). The peak voxel value and largest voxel cluster coincided, so this was chosen as the TMS target. He received 10 sessions per day of accelerated iTBS for 5 days, following an established dosing protocol for depression^44^, starting on a Thursday. Mild side effects included occasional headaches consistent with his baseline migraines and transiently feeling “a little hot and tired” for one hour. Clinically significant side effects included a period of intense sadness starting Friday afternoon, which lasted 4 hours and led to TMS being halted over the weekend. Treatment was resumed on Monday without recurrence of this symptom. Altogether, he received 50 treatments over 7 days.

After treatment, he reported dramatic improvement in PTSD symptom severity from “10/10” to “3/10.” This “3/10” rating was sustained at 1 week and at 4 weeks. At four weeks, 17 of 20 PTSD symptoms had improved (Fig. 6d) and his PCL-5 score had declined from 70/80 (severe PTSD) to 30/80 (no longer meeting PTSD threshold criteria).

## Discussion

We derived a brain circuit that is functionally connected to lesions that reduce the probability of developing PTSD. The circuit was reproducible, predicted PTSD outcomes in out-of-sample validation, and was not dependent on any single brain region or network. In patients without brain lesions, connectivity in this circuit was correlated with likelihood of PTSD symptoms and connectivity change in this circuit was correlated with reduction in PTSD symptoms following TMS. Finally, exploratory analyses suggest our circuit may parsimoniously explain diverse results of prior TMS trials focused on PTSD and fear.

These results build on prior work showing that lesions intersecting the amygdala reduce probability of developing PTSD^12^, but lesion location alone provided incomplete information. Intersection with the amygdala (or any single brain region) was less important than connectivity to a distributed brain network, as the PTSD circuit was robust even after excluding lesions that intersect the amygdala. The peak location in the circuit did not directly intersect any individual lesion in the whole sample, further demonstrating a network-level effect. While the circuit partly resembles the DMN, it also overlaps the limbic network, white matter, and subcortical regions.

Although perhaps unexpected, the mPFC was a stronger component of our circuit than the amygdala. mPFC is engaged during fear extinction in healthy controls^45,46^, while mPFC influences on the amygdala have been implicated in both fear conditioning and fear extinction^19,47,48^. Our lesion-based study complements prior work on human neuroimaging correlates of PTSD by increasing causal inference^9^, which can help identify therapeutic targets for neuromodulation^9^. Interpretation of our anatomical localization may also be guided by rodent studies^42,49^. Fear extinction localizes to the rodent infralimbic cortex, which is homologous to the human mPFC; by contrast, fear expression localizes to the prelimbic cortex, which is homologous to the human dorsal anterior cingulate cortex (dACC)^50^. Because the mPFC was a stronger part of our lesion-based PTSD circuit than dACC and aligned with outcomes in fear extinction trials, our results are consistent with prior rodent studies.

The intent of mapping this circuit was to choose where to stimulate with TMS, but it may also inform how to stimulate. Across multiple prior randomized trials, the effects of TMS to our circuit varied depending on whether excitatory or inhibitory protocols were used to modify fear conditioning or fear extinction. Our peak TMS target for PTSD was in the mPFC (MNI coordinates [−12,66,24]). We predict that this target could be used to improve PTSD by administering LF/cTBS with fear conditioning, HF/iTBS with fear extinction, or HF/iTBS with no task, as illustrated in our case report. This hypothesis is consistent with the convergence between our PTSD circuit and our previously-published anxiosomatic circuit, which was derived from HF without a task, and with animal models showing that HF to the mPFC can reverse stress-induced behavioral impairments^51^. It is also consistent with a recent trial showing that HF to the mPFC with a conditioning task was inferior to sham for PTSD, suggesting that the protocol may have inadvertently facilitated fear conditioning rather than extinction^52^.

Beyond the above peak target in the mPFC, our network-level approach also reveals additional extinction targets, consistent with emerging circuit models of PTSD^53^. Our target circuit includes the posterolateral right superior frontal gyrus (SFG), which may be an alternative excitatory target with an extinction condition (or no task). This DLPFC subregion is believed to regulate dysfunctional vmPFC-amygdala interactions in PTSD^53,54^. By contrast, more anterior SFG sites were negatively correlated with our PTSD circuit, and may be better inhibitory targets. These hypotheses were consistent with six prior trials across different TMS targets in the absence of a task, some of which were effective, some were ineffective, and some even exacerbated PTSD^17,55–59^. Thus, our results may unify prior seemingly-heterogeneous findings.

A strength of our study is demonstrating that the lesion-derived PTSD based circuit is abnormal in PTSD patients (without brain lesions) and correlated with PTSD improvement following TMS. This convergence is based on demonstrating similar topography, but the direction of rsFC abnormalities in PTSD patients is not directly interpretable. For example, it may seem counterintuitive that decreased rsFC within our PTSD circuit was associated with PTSD diagnosis but also with TMS-induced improvement. One might assume that TMS would need to increase or “normalize” rsFC within our circuit to improve PTSD. However, such discrepancies are common in functional neuroimaging, and highlight the difference between neuroimaging correlates and causation^9^. Just because reduced rsFC correlates with PTSD diagnosis, this does not mean that one should increase rsFC to improve PTSD. For instance, if decreased rsFC in our network is a compensatory response to PTSD, further decreasing rsFC might improve symptoms, and increasing rsFC would be counterproductive. For similar reasons, although TMS induced a decrease in rsFC, this does not constitute decreased brain activity. High frequency TMS, which is thought to increase activity and excitability, tends to decrease rsFC within the stimulated circuit^60^. Directly comparing correlative neuroimaging data with causal sources of information such as brain lesions may play an important role in better understanding the role of rsFC correlates of PTSD^9^.

Other strengths of our study include rigorous cross-validation, robustness across methods, and specificity to PTSD. The most important limitation is inability to compare different TMS sites and protocols head-to-head, particularly in context of fear-related tasks. Future prospective trials are needed to resolve this ambiguity. Our analysis also relies on heterogeneous data with multiple sources of noise. Many lesions were likely unrelated to PTSD, and some PTSD cases may have been unrelated to the event that caused the brain injury, particularly given that the specific nature of trauma and duration of illness were not recorded. By extension, our negative VLSM findings should not be interpreted as lack of a relationship between lesion location and PTSD, but rather inadequate power; LNM achieves greater statistical power by providing data for every patient at every voxel.^9^ In the non-lesion observational dataset, each patient had a 6-minute rsFC scan; this was standard when the data were collected, but recent guidelines recommend longer scans to reduce noise^61,62^. In the clinical trial dataset, the sample size was small and scan duration was only 8 minutes. We would expect these sources of noise to bias us towards a negative result, rather than consistent results across multiple datasets and modalities. However, future prospective studies are needed to address these limitations and directly test the current hypotheses.

In conclusion, we derived and cross-validated a brain circuit that is functionally connected to lesions that reduce probability of developing PTSD in Veterans. Increased connectivity in this circuit was associated with lower probability of PTSD symptoms and with TMS-induced reduction in PTSD severity. The peak superficial node in this circuit, the medial prefrontal cortex, may serve as a promising TMS target for PTSD.

## Methods

### Primary dataset

Data were analyzed from the Vietnam Head Injury Study (VHIS), which includes 252 participants who suffered severe traumatic injuries during the Vietnam War. 197 participants sustained penetrating head injury leading to a focal brain lesion, while 55 control participants sustained non-neurological injuries of comparable severity. Head CT scans were used to trace the boundaries of each lesion, which was transformed into MNI atlas space as described in Koenigs *et al*., 2008^12^. Seven participants (four in the head trauma group, three in the control group) were excluded due to incomplete data.

### Outcome measures

The SCID was chosen as the primary outcome because it has been used as the primary outcome in prior VHIS analyses and it reliably isolates the effect of PTSD versus other comorbidities^12,23^. For quantitative analysis, the “negative,” “subthreshold,” and “positive” categories were converted to scores of 0, 1, or 2, respectively. To assess whether brain lesions affected probability of developing PTSD symptoms, we used an unpaired t-test to compare PTSD scores in the brain lesion group versus the non-neurological control group.

### Lesion symptom mapping

First, we attempted to replicate prior findings by Koenigs et al^12^, which used the same dataset to show that patients with amygdala lesions had lower probability of developing PTSD. Using an unpaired t-test, we compared PTSD score in patients with amygdala lesions versus patients without amygdala lesions. Amygdala lesions were defined as any lesion that partly overlapped with the amygdala as defined by the Brainnetome atlas^28^.

Next, we used voxel lesion symptom mapping (VLSM)^29^ to identify specific voxels that were most associated with PTSD. At each voxel, we conducted an unpaired t-test to compare PTSD score in patients whose lesion overlapped with that voxel versus patients whose lesion did not overlap with that voxel. We tested for significance using split-half cross-validation as described below.

### Lesion network mapping

A normative human connectome database (n=1000) was used to estimate resting-state functional connectivity between each lesion location (n=193) and all other brain voxels. For each participant, this revealed a whole-brain “connectivity map” of that subject’s lesion. These 193 connectivity maps were compared with SCID scores using partial Pearson correlation, controlling for the effect of lesion size and depression severity. This yielded a whole-brain “circuit map” of connections correlated with PTSD diagnosis. This map was inverted such that positive values represent connections that reduce the probability of PTSD.

Prior work using the same dataset showed that amygdala lesions reduced the probability of developing PTSD^12^. To confirm that the current results were not driven by these amygdala lesions alone, we also repeated the analysis after excluding participants with amygdala lesions. To confirm that the results were not biased by subjects in the “subthreshold” category, the analysis was also repeated after excluding all subjects in this category. To confirm that the results were specific to PTSD rather than depression, the Beck Depression Inventory (BDI) was used as a covariate. To confirm that the results were not driven by other comorbidities, the analysis was also repeated after controlling for MacAndrews Alcoholism Risk score, mini-mental state examination score, total number of anxiety disorders on SCID, anxiety severity on the neurobehavioral rating scale, and continuous item-level scores on the CAPS.

### Statistical validation

Significance was assessed using split-half cross-validation with rigorous permutation-based statistical testing. These analyses were conducted separately for the VLSM technique and the lesion network mapping technique.

First, all 193 subjects were randomly split into two groups (n=97 and n=96). The lesion network mapping procedure and the VLSM procedure were repeated within each group. For each technique, this yielded a distinct map for each group. The similarity between these two maps was quantified using spatial correlation. The analysis was then repeated 1,000 times after randomly re-allocating the group assignments. We hypothesized that these spatial correlations would be significantly greater than zero.

We also used a permutation test to compare the resulting mean spatial correlation value to the spatial correlations expected by chance. The full analysis was repeated after randomly assigning each patient’s lesion network map to a different patient’s clinical outcome. This analysis was repeated 1,000 times to determine the distribution of spatial correlations expected by chance. We hypothesized that these spatial correlations would be significantly weaker than the mean spatial correlation for the real data.

The lesion network map includes both positive and negative correlations of lesions that reduce the risk of developing PTSD (Fig. S2). We hypothesized that lesion overlap with the positive component of the circuit would positively predict PTSD status, as the negative correlations may be epiphenomenal. To confirm this, we used both the positive and negative circuits to predict PTSD outcomes in another split-half analysis with 1,000 different random group re-allocations. First, we generated the positive and negative circuits in half of the dataset (n=97). For each of these two circuits, we took the sum of all voxel values that overlapped with each lesion in the other half of the dataset (n=96). This yielded a score for lesion overlap with the positive and negative circuits for each subject. A p-value was computed as the percentage of iterations that yielded a negative correlation. We hypothesized that the positive component of the circuit would predict PTSD status, while the negative component would not. We also repeated this analysis using spatial correlation of each lesion’s connectivity to the split-half circuit in lieu of spatial overlap of the lesion with the split-half circuit.

Of note, the above analyses were designed to test for significance of the overall network, as in our prior work on localizing TMS targets near the cortical surface for psychiatric disorders^16,30,33,63,64^. Some studies have also used LNM to map symptoms to individual brain regions rather than an entire network. To ensure that our results were robust to this method, we identified a peak location using permutation analysis of linear models with voxel-level family-wise error correction^65^. Additionally, a cluster centered on this location was identified using cluster extent thresholding (p_FWE_<0.05, detection p<0.001).

### Relevance to patients without brain lesions

We used two approaches to evaluate whether our results are relevant to PTSD patients without focal lesions. First, we compared our PTSD circuit to a meta-analytic region of interest (ROI) generated from Neurosynth, a tool that meta-analyzes the neuroimaging findings associated with any given search term^31^. We searched Neurosynth for “PTSD,” which yielded a binary map of voxels associated with that term. To quantify the overlap between our PTSD circuit (derived from lesions) and the Neurosynth ROI (derived from neuroimaging of pateints without lesions), we added all of the voxels in our unthresholded PTSD circuit that overlapped with the Neurosynth ROI. To determine if the degree of overlap was significantly stronger than chance, we repeated the full analysis 10,000 times after randomly permuting each patient’s lesion with a different patient’s clinical outcome. We hypothesized that the Neurosynth ROI would overlap more strongly with our PTSD circuit than with a randomly-permuted circuit. As a control, we also repeated this analysis using a Neurosynth ROI derived using the search term “depression” instead of “PTSD.”

In our second analysis, we computed individualized resting-state functional connectivity (rsFC) with our PTSD circuit in patients without brain lesions. This dataset included 62 Veterans with PTSD and 118 without PTSD. Other comorbidities included history of concussive traumatic brain injury (TBI) (n=55), subconcussive head injury (n=45), and depression (n=49). 47 subjects were healthy controls. PTSD diagnosis and comorbid generalized anxiety disorder were assessed via the SCID, and severity of individual PTSD symptoms was assessed using the Davidson Trauma Scale. Depression severity was assessed using the Beck Depression Inventory (BDI).

All patients completed a resting-state blood oxygen level dependent (BOLD) scan using a GE 750 scanner (3.75mm × 3.75mm × 3.75mm resolution, 6-minute duration). Data were pre-processed using CONN 18b, including atlas registration, frame realignment/motion correction, gray matter/white matter/cerebrospinal fluid regression, motion censoring, and temporal filtering (0.008 – 0.09 Hz).

Next, the positive component of our PTSD circuit was treated as a weighted seed for rsFC analysis. For each subject, rsFC was computed within this ROI. Binary predictors in the regression model included history of PTSD, history of TBI or subconcussive head injury, and active major depression (defined as BDI score of at least 20). We hypothesized that PTSD diagnosis would be independently associated with abnormal connectivity in the PTSD circuit.

### Anatomical and diagnostic specificity of individualized connectivity

To confirm that these results were anatomically specific to our PTSD circuit, we repeated the analysis using seven different resting-state brain networks as defined by Yeo et al., 2011^27^. We compared connectivity within each ROI and between each pair of ROIs, along with our PTSD circuit. This yielded 8 within-ROI comparisons and 28 pairwise ROI-ROI comparisons. One of these comparisons reflected connectivity within our PTSD circuit, while the other 35 comparisons were treated as control analyses. We hypothesized that PTSD would be more strongly associated with abnormal connectivity in the PTSD circuit than in any other ROI pair. We also hypothesized that this association would be statistically independent for the PTSD circuit versus the control ROIs – to test this, we repeated the analysis after controlling for the effect of all within- and between-ROI connectivity values. Because all values could not be assumed to follow identical distributions, all values were rank-transformed for this analysis.

To rule out the possibility that the analysis was biased by the choice of a *priori* seed, we conducted a whole-brain connectome-wide association study using multivariate distance matrix regression (MDMR), which does not depend on an a *priori* seed. Briefly, MDMR computes whole-brain connectivity of each voxel, quantifies the similarity between these whole-brain connectivity profiles across all 180 participants, and compares this value to PTSD status after controlling for other comorbidities^32^. This yielded a map of voxels that are different between PTSD patients and non-PTSD patients. We thresholded this map at p<0.01 and computed an odds ratio to assess the relative likelihood of these voxels being within our circuit. We compared this to the odds ratio for the seven Yeo networks described above.

We hypothesized that MDMR would yield a greater percentage of abnormal voxels inside our circuit than outside our circuit. To test this hypothesis, we recomputed these values after randomly permuting each participant’s clinical outcomes with a different participant’s neuroimaging results in both datasets, and computed a p-value as the percentage of instances in which the real data were stronger than the permuted data. To confirm that this result was not biased by the choice of MDMR p-value threshold, we also repeated the analysis with thresholds of p<0.001 and p<0.05.

### Modulation of the circuit with TMS

To assess whether this circuit is relevant for TMS response, we evaluated 20 Veterans who received resting-state fMRI scans as part of a randomized, double-blind clinical trial of TMS for PTSD (n=10 active, n=10 sham). Patients received 10 daily sessions of intermittent theta burst stimulation targeted to the right DLPFC using the Beam F4 procedure (80% active motor threshold, 1800 pulses, 9.5 minutes)^7^. Stimulation was delivered using a Magstim 2 + 1 TMS system (Magstim, UK). Sham was delivered using a coil that applies a superficial pulse to produce a similar scalp sensation, but does not penetrate into the brain^7^. Participant demographics are listed in Table S2.

All patients completed a resting-state BOLD scan (3 × 3 × 3 mm resolution, 8-minute duration). PTSD severity was measured using the PTSD Checklist for DSM-5 (PCL-5), depression severity was measured using the Inventory for Depressive Symptomatology (IDS), and anxiety severity was measured using the state-trait anxiety inventory (STAI). We computed individualized rsFC within our circuit before and after the treatment course.

We hypothesized that connectivity change within our circuit would be associated with percentage improvement in PTSD, independently of improvement in depression. To test this hypothesis, we used a linear mixed model to assess for a group × connectivity change interaction on PCL-5 change after controlling for IDS change. We also tested for specificity as above.

### Explaining variance in published TMS trials

Our targeting atlas addresses the optimal stimulation locations, but not the optimal protocol. Some TMS protocols are more likely to be excitatory, while others are more likely to be inhibitory^66^, although this can vary between individuals^67^. The effects of excitatory versus inhibitory protocols also differ depending on whether they are paired with a fear extinction or fear conditioning task. We investigated this in the published literature. Because our circuit was derived from lesions (presumed to be inhibitory) that improved outcomes after a traumatic event (fear conditioning cue), we hypothesized that fear would be reduced by inhibitory stimulation with a fear conditioning task, and would be worsened by inhibitory stimulation with a fear extinction task. Conversely, we hypothesized the opposite effects with excitatory stimulation or with inhibitory stimulation to the anti-correlated circuit.

We searched PubMed for (“transcranial magnetic stimulation” or “TMS”) and (“fear”). We included randomized, controlled studies in which repetitive TMS was paired with either a fear-inducing cue or a fear extinction paradigm. We excluded studies with less than 10 participants per group and studies that stimulated outside of the prefrontal cortex. Effect sizes were treated as positive if they supported our hypothesis, or negative if they supported the null hypothesis.

### Proposed TMS targets and protocols

To develop a TMS “targeting atlas,” we computed whole-brain normative connectivity to our PTSD circuit. The peak positive and negative values in this atlas may represent prospective TMS targets. Since TMS is not able to directly access deep regions such as the amygdala and anterior cingulate cortex, we compared this circuit to common TMS targets that have been used in different clinical trials for depression, including the mPFC, the “5cm” and EEG F3/F4 targets, and the “6 cm” and Beam F3/F4 targets^68^. We chose the target which showed the strongest association with this map as a prospective TMS target for PTSD.

Next, we compared our PTSD targeting atlas to our previously-published “anxiosomatic” brain circuit. Stimulation of this circuit has been associated with improvement in anxious and somatic symptoms of depression, such as sexual dysfunction, insomnia, and irritability^16^. We used spatial correlation to assess similarity of the anxiosomatic circuit to our PTSD circuit and tested for significance using permutation testing as above.

Finally, we compared our PTSD targeting atlas to prior clinical trials of TMS for PTSD with no psychological task. We searched PubMed for (“TMS” or “transcranial magnetic stimulation”) and (“PTSD” or “posttraumatic stress” or “post-traumatic stress”). As above, we excluded studies with less than 10 participants per group or studies in which the target could not be localized. Three studies were excluded for this reason because the target selection procedure was not described (Jiang 2023), an atypical target selection procedure was used that has never been translated into MNI space (Watts 2012), or multiple conflicting target selection procedures were described (Ahmadizadeh 2018). To avoid circularity, we also excluded the TMS clinical trial that had already been used for assessment of TMS-induced connectivity change (Philip 2019).

### Case of prospective TMS to the circuit

We treated an individual patient using accelerated iTBS to our circuit. He received a baseline resting-state fMRI scan with three 6.5 minute runs (total 22-minute acquisition), 3.4 × 3.4 × 4 mm spatial resolution, and 2.15-sec TR. The target was identified using seed-based connectivity to our PTSD circuit, followed by manual review of the resulting images. Treatment included including 10 sessions per day of 1800 pulses, spaced by 50 minutes, at 120% of motor threshold.

## Data Availability

The functional connectivity data employed in this study are available online through the Harvard Dataverse at: https://doi.org/10.7910/DVN/ILXIKS. Individual patient data cannot be shared publicly because imaging and psychiatric scales may contain identifiable information. Multiple datasets from multiple institutions are reported in this manuscript; data are available upon reasonable request with an approved data use agreement with the institution at which each dataset was collected. Questions regarding the Vietnam Head Injury Study can be directed to Dr. Jordan Grafman (jgrafman@northwestern.edu).
